# Cyclin E involved in early stage carcinogenesis of esophageal adenocarcinoma by SNP DNA microarray and immunohistochemical studies

**DOI:** 10.1186/1471-230X-14-78

**Published:** 2014-04-17

**Authors:** Zhongren Zhou, Santhoshi Bandla, Jiqing Ye, Yinglin Xia, Jianwen Que, James D Luketich, Arjun Pennathur, Jeffrey H Peters, Dongfeng Tan, Tony E Godfrey

**Affiliations:** 1 Departments of Pathology and Laboratory Medicine, University of Rochester, Rochester, 601 Elmwood Ave, Box 626, Rochester, NY 14642, USA; 2 Departments of Surgery, University of Rochester, Rochester, NY, USA; 3 Biostatistics and Computational Biology, University of Rochester, Rochester, NY, USA; 4 Biomedical Genetics, University of Rochester, Rochester, NY, USA; 5 Department of Cardiothoracic Surgery, University of Pittsburgh Medical Center, Pittsburgh, PA, USA; 6 Department of Pathology, The University of Texas MD Anderson Cancer Center, Houston, TX, USA; 7 Department of Surgery, Boston University School of Medicine, Boston, MA, USA

**Keywords:** Esophageal adenocarcinoma, Cyclin E, Amplification, High expression, Barrett’s esophagus, SNP DNA microarray, Biomarker, Overall survival

## Abstract

**Background:**

Cyclin E is a cell cycle regulator which is critical for driving G1/S transition. Abnormal levels of cyclin E have been found in many cancers. However, the level changes of cyclin E in esophageal adenocarcinoma and its precancerous lesion have not been well studied. Here, we focus on the gene amplification and expression of cyclin E in these lesions, and aim to ascertain the relationship with clinicopathological characteristics.

**Methods:**

Genomic DNA was analyzed from 116 esophageal adenocarcinoma and 26 precancerous lesion patients using Affymetrix SNP 6.0 arrays. The protein overexpression of cyclin E was also detected using immunohistochemistry from tissue microarrays containing esophageal adenocarcinoma and precancerous lesions. Patient survival and other clinical data were collected and analyzed. The intensity and percentage of the cyclin E expressing cells in tissue microarrays were scored by two pathologists. Fisher exact tests and Kaplan-Meier methods were used to analyze data.

**Results:**

By genomic analysis, cyclin E was amplified in 19.0% of the EAC samples. By immunohistochemistry, high expression of cyclin E was observed in 2.3% of squamous mucosa tissues, 3.7% in columnar cell metaplasia, 5.8% in Barrett’s esophagus, 19.0% in low grade dysplasia, 35.7% in high grade dysplasia, and 16.7% in esophageal adenocarcinoma. The differences in cyclin E high expression between neoplastic groups and non-dysplasia groups are statistically significant (*p* < 0.05). The prognosis for patients with high cyclin E expression appeared slightly better than for those with low cyclin E expression although this was not statistically significant (p = 0.13).

**Conclusions:**

The expression of cyclin E significantly increases from non-dysplasia esophageal lesion to low and high grade dysplasia, suggesting that cyclin E plays an important role in the early stage of carcinogenesis. Importantly, cyclin E is also amplified and highly expressed in a subset of esophageal adenocarcinoma patients, but this increase is not associated with worse prognosis.

## Background

The incidence of esophageal adenocarcinoma (EAC) has increased approximately 600% in the US and other Western Countries over the last 30 years [1]. EAC tends to be diagnosed late with most patients in locally advanced or metastatic disease. Consequently, the overall prognosis for patients with EAC is very poor at approximately 15%, with 5-year overall survival. More than 50 percent of patients have either unresectable tumors or radiographically visible metastases at the time of diagnosis [2]. Identification of early biomarkers with high sensitivity and specificity will provide physicians with valuable information for surveillance, diagnosis, prognosis, and possible treatment options for esophageal adenocarcinoma. Previous studies have suggested that esophageal adenocarcinoma develops in the following order: normal esophageal epithelium, reflux esophagitis, Barrett’s esophagus (BE), dysplasia, and finally esophageal adenocarcinoma [3]. During these events, a series of genetic and epigenetic aberrations contributes to the carcinogenesis, which will be potential biomarkers for early screening, surveillance and treatment of the dysplasia and adenocarcinoma.

Cyclin E, an activating subunit of cyclin dependent kinase 2 (*CDK2*), is encoded by human cyclin E1 gene (*CCNE1*) on chromosome 19q12-13. Cyclin E plays a key role to promote G1 cell cycle transition to S-phase. The oncogenic activity of cyclin E is involved in multiple functions including a regulatory network comprised CDK inhibitors, the p53 and FBW7 tumor suppressor pathways, signal transduction pathways, controlling cell cycle progression, and microRNAs [4,5]. Genetic and pharmacologic targeting of the cyclin E-CDK-2 complex resulted in marked growth inhibition of lung cancer cells [6], suggesting a potential chemotherapeutic approach for lung cancer. In breast cancer, the depletion of cyclin E by siRNA promoted apoptosis of cyclin E overexpressing cells and blocked their proliferation, transformation phenotype and tumor growth in nude mice. Liang and colleagues concluded that cyclin E may serve as a novel and effective therapeutic target [7]. In addition, the amplification and overexpression of cyclin E have been reported in a variety of cancers including breast [7,8], lung [9], ovarian [10], stomach [11,12], colorectal [13,14], bladder [15], endometrial carcinoma [16] and thyroid [17]. In the esophagus, a few studies found cyclin E amplification and overexpression in esophageal adenocarcinoma and precancerous lesion in small samples [18-21].

The cyclin E expression was first reported in low-grade dysplasia (2/21), high grade dysplasia (3/17), adenocarcinoma (5/35) and Barrett’s esophagus (43%) in 60 samples by an immunohistochemistry [21,22]. Cyclin E gene amplification in esophageal adenocarcinoma was also confirmed in 13.8% (9/65) [19] and 12.6% (11 of 87) [20] in esophageal adenocarcinoma by quantitative PCR molecular analysis [19,20]. However, the sample size of previous studies is small and the results were not consistent. In addition, the relationship between high expression of cyclin E or gene amplification and the patient survival is unknown.

In the current study, we (i) used high resolution SNP DNA microarray to study cyclin E amplification in the large scale of esophageal adenocarcinoma and precancerous lesions; (ii) used immunohistochemical method to confirm the high expression of cyclin E in a larger number of esophageal adenocarcinoma and precancerous lesions; and (iii) studied the association of cyclin E amplification and high expression with patients’ overall survival and clinicopathological features.

## Methods

### Patients for Affymetrix SNP 6.0 analysis

Frozen tumors were obtained from 116 patients undergoing esophagectomy at the University of Pittsburgh Medical Center, Pittsburgh, PA between 2002 and 2008. Patient age ranged from 43–88 and the cohort consisted of 95 males and 21 females. Final pathologic stages were stage I (28), stage II (31), stage III (50) and stage IV (7). All tumor specimens were evaluated by a pathologist and were determined to be >70% tumor cell representation. Only 112 specimens were used for survival analysis as we excluded 4 peri-operative chemotherapy patients.

Frozen Barrett’s esophagus (intestinal metaplasia: n = 26) and esophageal columnar cell metaplasia (metaplasia without goblet cells; n = 25) biopsy tissues were obtained from patients undergoing endoscopy at the University of Rochester Medical Center from 2008 to 2012. All pathologic diagnoses were evaluated by pathologists. All studies were approved by research subjects review board at University of Pittsburgh and University of Rochester.

### Affymetrix SNP 6.0 analysis

Genomic DNA was isolated using the QiaAmp DNA Mini Kit (Qiagen, CA) and 600 mg was used for labeling and array hybridization at the SUNY Upstate Medical University microarray core facility (Syracuse, NY) using kits and protocols provided by Affymetrix. Data analysis was performed using Nexus 6.0 Copy Number Analysis software (Biodiscovery, Inc. CA). Log_2_ DNA copy number ratios for the tumor and pre-neoplastic samples were generated in reference to a baseline file created using DNA from normal esophageal mucosa from a subset (n = 15) of the Pittsburgh patient cohort. Data was segmented using the SNP-Rank segmentation algorithm with a minimum of 8 probe sets and significance threshold of p-value of 10^−6^. Log_2_ copy number threshold for gains were set at +0.15 (~2.2 copies) while high level gains were set at +0.5 (~2.8 copies). More information on this patient cohort and a comprehensive genomic analysis of these tumors is to be published by Dulak et al [23]. Microarray data on this cohort has been submitted to the Gene Expression Omnibus (GSE36460) with an online link (http://www.ncbi.nlm.nih.gov/geo/query/acc.cgi?acc=GSE36460).

### Construction of tissue microarray

Tissue microarrays, containing 34 cases of Barrett’s esophagus (BE), 81 cases of columnar cell metaplasia (CCM), 86 cases of squamous epithelium (SE), 21 cases of low grade dysplasia (LGD), 14 cases of high grade dysplasia (HGD), and 117 cases of esophageal adenocarcinoma (EAC), were constructed from the representative areas of formalin-fixed specimens collected between 1997–2005 in the Department of Pathology and Laboratory Medicine, University of Rochester Medical Center/Strong Memorial Hospital, Rochester, New York. Five-micron sections were cut from tissue microarrays and were stained with H&E to confirm the presence of the expected tissue histology within each tissue core. Additional sections were cut for immunohistochemistry analysis.

### Patients for tissue microarrays

All 117 patients with EAC used for the tissue microarray construction were treated with esophagectomy at Strong Memorial Hospital/University of Rochester from 1997 to 2005. These patients included 105 males and 12 females. The patients’ ages ranged from 34 to 85 years (Table 1). The follow-up period after esophagectomy ranged from 0.3 to 142 months with a mean of 39 months.

**Table 1:** Distribution of patients in Age and Sex with esophageal adenocarcinoma and precancerous lesion using immunohistochemical study

**Diagnosis**	**Total**	**Female**	**Male**	**Age**
Adenocarcinoma	117	12	105	65
High grade dysplasia	14	2	12	67
Low grade dysplasia	21	0	21	71
Barrett’s esophagus	34	4	30	67
Columnar cell metaplasia	81	7	74	64
squamous epithelium	86	19	67	65

### Immunohistochemistry

Tissue sections from the tissue microarray were deparaffinized, rehydrated through graded alcohol, and washed with phosphate buffered saline. Antigen retrieval for cyclin E was performed by heating sections in 99°C water bath for 40 minutes. After endogenous peroxidase activity was quenched and nonspecific binding was blocked, ready-to-use mouse monoclonal antibody anti-cyclin E (Santa Cruz, CA) was incubated at room temperature for 30 minutes. The secondary antibody (Flex HRP) was allowed to incubate for 30 minutes. After washing, sections were incubated with Flex DAB Chromogen for 10 minutes and counterstained with Flex Hematoxylin for 5 minutes. A colon adenocarcinoma with known cyclin E high expression served as positive control. Negative control was performed by replacing the anti-cyclin E antibody with the normal serum. Several tissue cores were falloff glass during this processing.

### Scoring of immunohistochemistry

**Figure 1:**
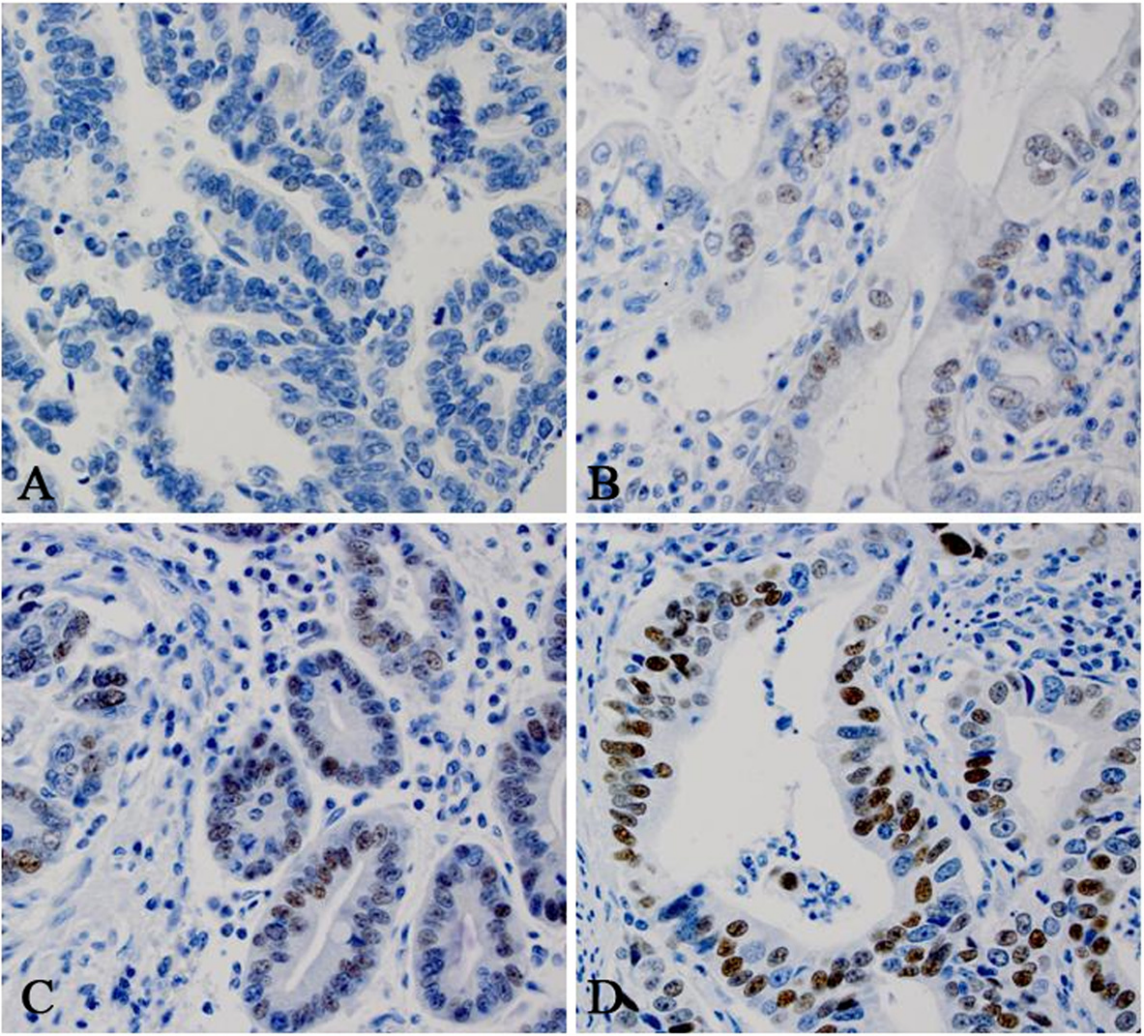
**The intensity of cyclin E immunohistochemical study with nuclear staining. A**. 0; Negative or very week intensity of cyclin E nuclear stain in one EAC sample; **B**. 1+: weak intensity of cyclin E nuclear stain in one EAC sample; **C**. 2+: moderate intensity of cyclin E nuclear stain in one EAC sample; and **D**. 3+: strong intensity of cyclin E nuclear stain in one EAC sample.

All sections were reviewed independently by JY and ZZ blinded to all clinical and pathologic information. Discordant cases were reviewed by both JY and ZZ and a final consensus was reached. The percentage (0-100%) of the cells with positive nuclear staining was recorded. The intensity of cyclin E nuclear staining was graded as 0, 1+, 2+, or 3+. No nuclear stain or positive nuclear stain in less than 10% was defined as 0 (Figure 1A); weakly nuclear stain in 10% or more cells was defined as 1+ (Figure 1B); relatively strong nuclear stain in 10% or more cells was defined as 2+ (Figure 1C); very strong nuclear stain in 10% or more cells was defined as 3+ (Figure 1D). Cyclin E protein was considered highly expressed if 10% or more of cells stained with a moderate to strong intensity (2+ and 3+, respectively) (Figure 1).

### Statistical analysis

All the descriptive statistics in this study were presented as means. A *P*-value of less than 0.05 was considered statistically significant. The univariate analysis with cyclin E was conducted first and then followed with a multivariate analysis, including age, gender, and clinical covariates: lymph node metastasis and tumor stage. Chi-square and Fisher exact tests were used as appropriately to compare cyclin E positivity rates from columnar cell mucosa, dysplasia to adenocarcinoma. To evaluate the influence of high expression of cyclin E in esophageal adenocarcinoma, comparative risk analysis using the Kaplan-Meier method cooperated with the log-rank test was performed with cyclin E amplified and non-amplified groups. All the statistical analyses were conducted with SAS 9.3 software (SAS Institute Inc., Cary, NC).

## Results

### Genomic analysis of cyclin E amplification

**Figure 2:**
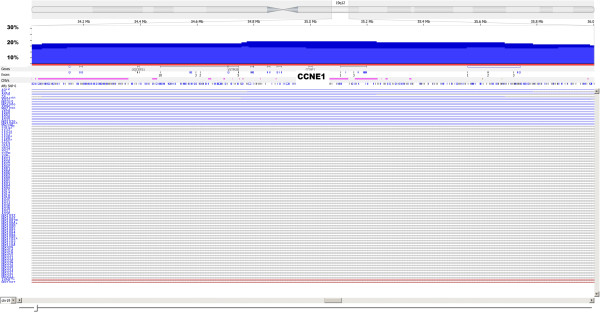
**Frequency histogram showing amplification of the *CCNE1 *locus at chromosome 19q12-13 in 116 esophageal adenocarcinoma samples using high density copy number SNP microarrays.** This locus is amplified in 22/116 (19.0%) cases in this patient cohort, approximately half of which are considered high copy number amplification events.

Analysis of 116 EAC specimens using high density copy number microarrays revealed amplification of *CCNE1* in 19.0% (22/116) (Figure 2). In this cohort, the median overall survival of patients with *CCNE1* amplification was approximately 20 months compared with 25 months for those without amplification (*p =* 0.22). *CCNE1* amplification was not observed in Barrett’s esophagus (0/26) or columnar cell metaplasia specimens (0/25).

### Immunohistochemical characteristics and analysis of cyclin E expression

**Figure 3:**
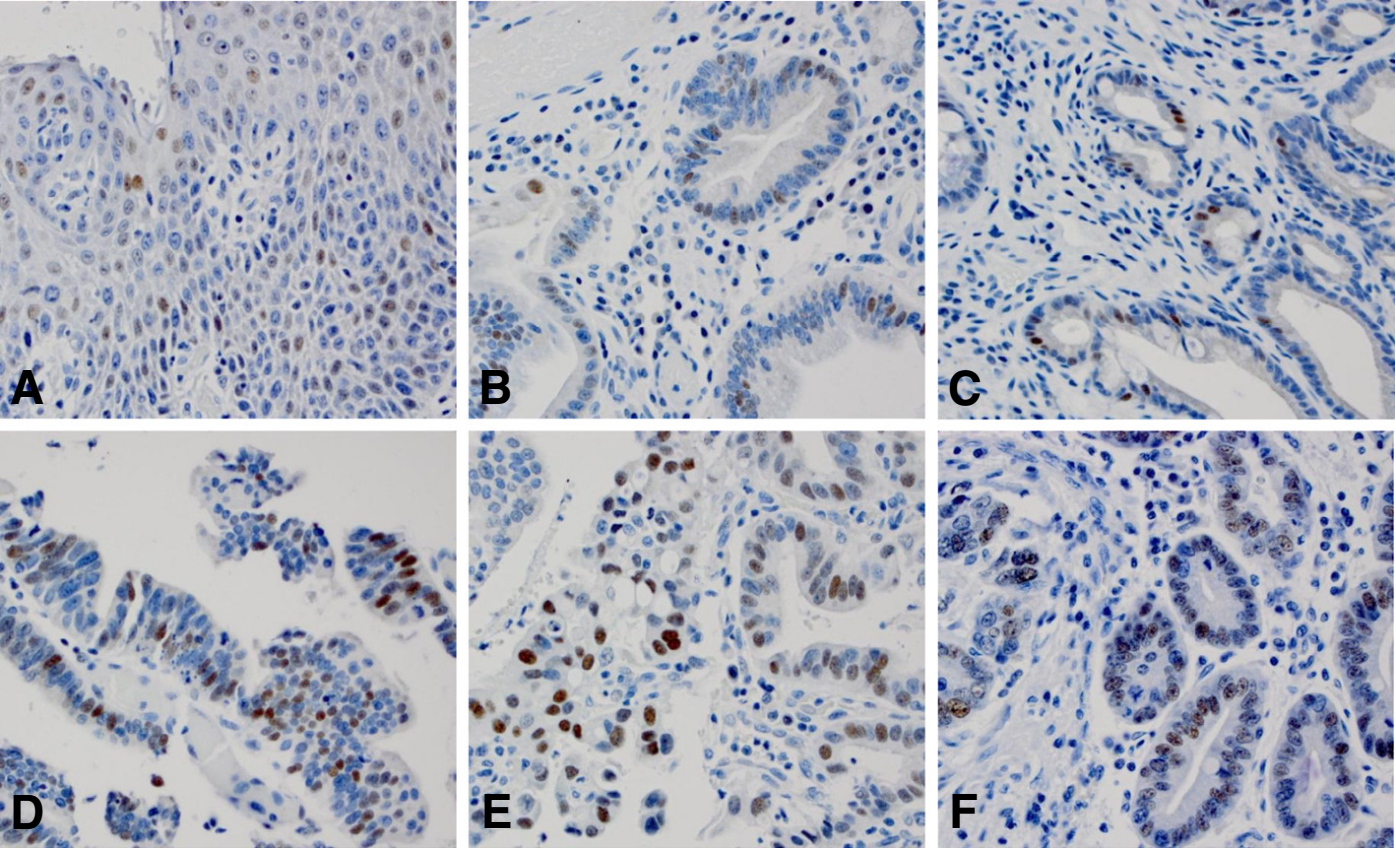
**High expression of cyclin E in various histologic types by immunohistochemical studies.** Cyclin E immunostain shows weakly nuclear stain in squamous mucosa **(A)**, columnar cell metaplasia **(B)** and Barrett’s esophagus **(C)**. Cyclin E shows strong nuclear stain in low grade dysplasia **(D)**, high grade dysplasia **(E)** and adenocarcinoma **(F)**.

By immunohistochemical analysis, high expression of cyclin E was observed in 2.3% of normal squamous mucosa (2/86), 3.7% in columnar cell metaplasia (3/81), 5.8% in Barrett’s esophagus (2/34), 19.0% in low grade dysplasia (4/21), and 35.7% in high grade dysplasia (5/14). In esophageal adenocarcinoma high cyclin E expression was observed in 16.7% (19/114) of cases. This was not statistically significantly different from high grade dysplasia. Qualitatively, we observed that normal squamous mucosa and columnar cell metaplasia usually have weak, focal staining whereas high grade dysplasia or adenocarcinoma have strong, diffuse staining (Table 2 and Figure 3).

**Table 2:** High expression of cyclin E in esophageal adenocarcinoma, low and high dysplasia, Barrett’s esophagus, columnar cell metaplasia and squamous cells

**Diagnosis**	**Non-/low expression (n; %)**	**High expression (n; %)**	**Total**
squamous epithelium	84 (97.7)	2 (2.3)	86
Columnar cell metaplasia	78 (96.3)	3 (3.7)	81
Barrett’s esophagus	32 (94.2)	2 (5.8)	34
Low grade dysplasia	17 (81.0)	4 (19.0)	21
High grade dysplasia	9 (64.3)	5 (35.7)	14
Adenocarcinoma	95 (83.3)	19 (16.7)	114

Chi-square and Fisher exact tests were used to compare cyclin E percentages among all various histological groups including squamous epithelium, columnar cell mucosa, Barrett’s esophagus, low- and high-grade dysplasia, and adenocarcinoma. The differences of cyclin E high expression between all neoplastic groups (including EAC, HGD and LGD) and non-dysplasia groups (including CCM and SE) are statistically significant (p < 0.05) (Table 3). No significant difference is identified among neoplastic groups. In addition, no significant difference of cyclin E high expression is identified between squamous mucosa and columnar cell metaplasia. Barrett’s esophagus group is only significantly different from high grade dysplasia (Table 3).

**Table 3:** **Comparison of the frequency of cyclin E high expression between various groups by Fisher exact test (*p *** **value)**

**Group1**	**Group 2**	*p*
SE	CCM	0.3060
SE	BE	0.2496
SE	LGD	0.0121*
SE	HGD	0.0014*
SE	AC	0.0011*
CCM	BE	0.3119
CCM	LGD	0.0277*
CCM	HGD	0.0014*
CCM	AC	0.0051*
BE	LGD	0.1158
BE	HGD	0.0153*
BE	AC	0.0890
LGD	HGD	0.1697
LGD	AC	0.2192
HGD	AC	0.0538

*The frequency of cyclin E high expression shows significantly different between these pairs.

*CCM*, Columnar cell metaplasia; *BE*, Barrett’s Esophagus; *LGD*, Low grade dysplasia (LGD); *HGD*, High grade dysplasia; *EAC*, Esophageal adenocarcinoma; *SE*, Squamous epithelium.

### Survival analysis of cyclin E high expression in EAC

**Figure 4:**
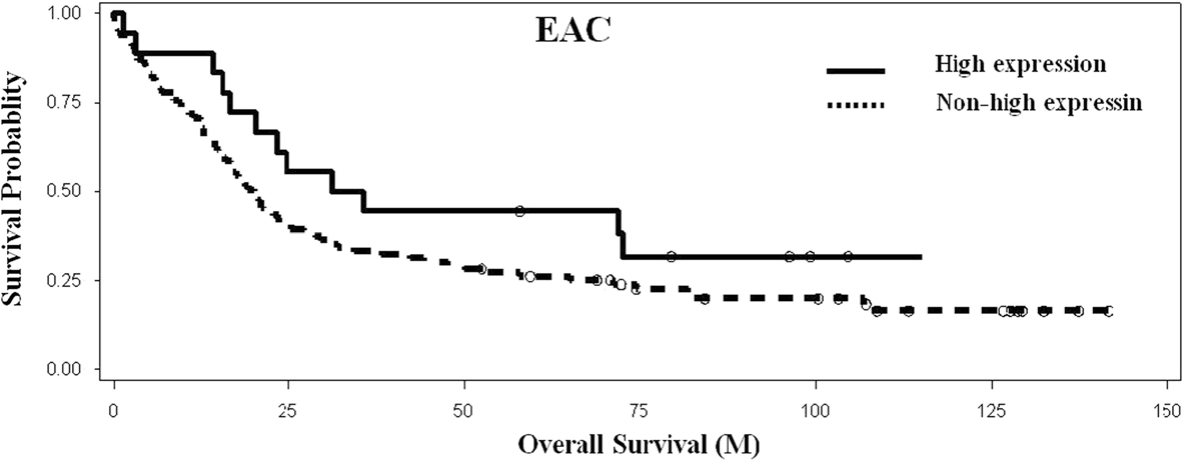
**Kaplan-Meier analysis of overall survival associated with high cyclin E expression in esophageal adenocarcinoma.** No significant association of overall survival with cyclin E high expression (*p =* 0.13) in 117 EAC patients.

Kaplan-Meier analysis and the log-rank test were used to calculate the effect of the cyclin E high expression in patients with EAC on survival. The mean overall survival in the cyclin E high expression group was 42 months, while that in the group without high cyclin E expression was 38 months. The log-rank test showed a trend towards better overall survival in the high-cyclin E group but this was not statistically significance (*p* = 0.13, Figure 4).

Multivariate survival analysis of clinical covariates including age, gender, histologic grade, and stage in EAC, found that age, differentiation and stage (*p* < 0.05) have strong association with patient survival, but gender (*p* = 0.66) was not significantly associated with patients’ survival in EAC.

### Correlation of cyclin E high expression and clinicopathological characteristics

The correlation of high cyclin E expression with clinicopathological features was analyzed. High expression of cyclin E is not associated with age, gender, stage, differentiation and lymph node metastasis (data not shown).

## Discussion

In this study we found that cyclin E shows a significantly higher frequency of high expression in neoplastic lesions (low- and high-grade dysplasia or adenocarcinoma) compared to non-dysplastic tissues (Barrett’s esophagus, columnar cell metaplasia and squamous epithelium). With SNP DNA microarray study, the amplification of cyclin E was also present in esophageal adenocarcinoma, but was not identified in Barrett’s esophagus and columnar cell metaplasia. In addition, we found that high expression of cyclin E may be associated with better prognosis although this did not reach statistical significance.

Sarbia et al. first reported that the expression of cyclin E in esophagus tissues in small samples was present in 0 of 24 SE (0.0%), 2 of 21 LGD (9.5%), 3 of 17 HGD (17.6%), and 5 of 35 CA (14.3%) [22]. In our study, cyclin E shows similar frequency of high expression in 16.7% esophageal adenocarcinoma (19/114), but a higher frequency of expression in high grade dysplasia (35.7%) and low grade dysplasia (19.0%) compared to their study. In addition, we found that cyclin E is highly expressed with lower rates at 5.8% in Barrett’s esophagus (2/34), 3.7% in columnar cell metaplasia (3/81), and 2.3% in squamous mucosa (2/86). Umansky et al. also reported the expression of cyclin E (43%), p16 (73%), p21 (88%), p27 (95%), and cyclin D1(47%) in Barrett’s esophagus, which was down-regulated by acid suppression of proton pump inhibition (PPI). However, no amplification or deletion was identified by Southern blot analysis [21]. This suggests that episodes of acid reflux might trigger proliferation and inhibit programmed cell death signaling pathways. In our study, no amplification was identified in Barrett’s esophagus (0/26) and columnar cell metaplasia (0/25) by SNP DNA microarray method. However, high expression of cyclin E (5.8%) in BE is significant lower than that in Umansky’s study (43%). The mechanism is unclear how cyclin E is highly expressed in BE and columnar cell metaplasia without the amplification. Cyclin E amplification was observed at 13.8% (9/65) [19] and 12.6% (11 of 87) [20] in esophageal adenocarcinoma, which is lower than our SNP DNA microarray data (19.0%).

Cyclin E was reported to be expressed in precancerous lesion of colon adenocarcinoma [14,24,25]. Expression of cyclin E has been shown in 25% of colorectal adenomas, the most important precursor lesions of colorectal carcinoma [24]. With 1,2-dimethyl-hydrazine dihydrochloride (DMH)-induced rat colon adenocarcinoma, cyclin E expression was detected in 87.5% of the adenomas and in 92.3% of the adenocarcinomas [25]. Hur and colleagues also found that cyclin E expression both in the mRNA and protein levels was present in normal colonic mucosa, adenomas and adenocarcinomas. There was a significant difference in the degree of expression of cyclin E between normal mucosa and adenomas, but there was not a significant difference between adenomas and adenocarcinomas. They indicated that cyclin E plays an important role during the multistage process of rat colon carcinogenesis, especially at a relatively early stage [25]. In human samples, the increase of cyclin E expression also was reported in colon mucosa. The median of cyclin E expression significantly increased in normal through hyperplastic and adenomatous tissues and slightly decreased in adenocarcinoma of colon samples [14], which confirmed the finding in the rat model and proved that the expression of cyclin E promoted abnormal proliferation of cells during colorectal carcinogenesis [14]. In the esophagus, our data and previous studies also showed that the high expression of cyclin E significantly increased from non-dysplasia group (normal squamous epithelium, columnar cell metaplasia) to neoplastic group (low and high grade dysplasia). The high expression of cyclin E reached its peak in high grade dysplasia and decreased in adenocarcinoma. Our findings in the esophagus agree to the previous studies in colon. High expression of cyclin E may play an important role in early stage of carcinogenesis in esophagus and could be a potential targeted marker to early interfere with cancer progress and stratify high risk patients with precancerous lesion for close surveillance.

Cyclin E and paired CDK 2 are important antineoplastic targets in oncology. siRNA treatment significantly reduced *CCNE1* or *cyclin E-CDK-2* complex expression and significantly inhibited cell growth in *CCNE1*-expressing cells, suggesting that *CCNE1*-targeted therapy may benefit ovarian, breast and lung cancer patients with *CCNE1* amplification [6,7,10]. In addition, cyclin E siRNA synergistically enhanced the cell killing effects of doxorubicin in cell culture and suppressed the tumor growth in mice. They concluded that cyclin E may serve as a novel and effective therapeutic target [7]. Our study showed both amplification and high expression of cyclin E in esophagus precancerous lesion and adenocarcinoma, suggesting the further study of potential effect in the inhibition of cyclin E expression for target therapy of esophageal precancerous lesion.

Amplification and high expression of cyclin E were reported to relate with poor prognosis in many different tumors [8-10,12,14-16,26]. In meta-analysis of lung non-small cell carcinoma from fourteen studies (2606 cases) [27], cyclin E over-expression was found to be a strong predictor of poor prognosis in lung carcinoma patients (HR: 1.38, 95% CI: 1.07-1.79; P = 0.014). In ovarian cancer, the amplification was identified in 18 (20%) of 88 ovarian carcinoma, which was significantly correlated with shorter disease-free survival and overall survival [10]. In gastric [11] and colorectal adenocarcinoma [28], overexpression of cyclin E was a potential prognostic markers. It is surprising to find both amplification and high expression of cyclin E in esophageal adenocarcinoma in our study were not significantly associated with patient overall survival, even with a little better overall survival rate with high expression of cyclin E. The controversial data for the prognosis was reported in the colon [29,30], ovary [31], stomach [11,12] and lung [9]. In the esophagus, similar to the cyclin E study, we recently found that HER2 amplification and expression were associated with better but not significantly better prognoses [32], which is confirmed by a Mayo clinical study [33]. They further proved that HER2 positivity was significantly associated with a better survival. Therefore, the function of oncogene may play different roles in various organs or tumors. Furthermore, our findings needs to be confirmed by different studies since the cyclin E expression and amplification are associated with the sensitivity of methods, race of patients, location of tumors and pre-operative neoadjuvant therapy.

## Conclusions

The high expression of cyclin E significantly increases from non-dysplasia esophageal lesion, to low and high grade dysplasia. It implies that cyclin E may play an important role in early stage of carcinogenesis and could be a potential marker for a target therapy of precancerous lesion. In addition, the amplification and high expression of cyclin E are associated with a better prognosis, but not statistically significant.

## Competing interests

The authors declare that they have no competing interests.

## Authors’ contributions

ZZ and TG: Designing the project; ZZ: Write the paper. ZZ, TG, JQ and DT: editing the paper and consultation for the project. ZZ and JY: Scoring all IHC slides from TMA; YX: Involving data analysis; TG and SB: Analyzing SNP DNA microarray data; JP, AP, and DT: Collecting the clinicopathological information and tissue. All authors read and approved the final manuscript.

## Pre-publication history

The pre-publication history for this paper can be accessed here:

http://www.biomedcentral.com/1471-230X/14/78/prepub
